# Uncovering the Role of PhzC as DAHP Synthase in Shikimate Pathway of *Pseudomonas* *chlororaphis* HT66

**DOI:** 10.3390/biology11010086

**Published:** 2022-01-06

**Authors:** Songwei Wang, Dongliang Liu, Muhammad Bilal, Wei Wang, Xuehong Zhang

**Affiliations:** 1 State Key Laboratory of Microbial Metabolism, School of Life Sciences and Biotechnology, Shanghai Jiao Tong University, Shanghai 200031, China; songwei2211@sjtu.edu.cn (S.W.); weiwang100@sjtu.edu.cn (W.W.); 2 CAS Key Laboratory of Computational Biology, Shanghai Institute of Nutrition and Health, University of Chinese Academy of Sciences, Chinese Academy of Sciences, Shanghai 200031, China; liudongliang2018@sibs.ac.cn; 3 School of Life Science and Food Engineering, Huaiyin Institute of Technology, Huaian 211600, China; bilaluaf@hotmail.com

**Keywords:** *Pseudomonas* *chlororaphis*, PhzC, shikimate pathway, phenazine-1-carboxamide

## Abstract

**Simple Summary:**

This study investigated PhzC, one essential 3-Deoxy-D-arabino-heptulosnate-7-phosphate (DAHP) synthase that catalyzes the first step of the shikimate pathway in *Pseudomonas* *chlororaphis*. We identified and characterized *phzC*, which is different from the reported DAHP synthase encoding genes *aroF*, *aroG* and *aroH* in *E*. *coli*. PhzC accounts for approximately 90% of the total DAHP synthase activities in *P*. *chlororaphis* and it plays the most critical role in four DAHP synthases in the shikimate pathway. Moreover, the results showed that *phzC* in *P*. *chlororaphis* HT66 is not sensitive to feedback inhibition. This study demonstrated that PhzC is essential for phenazine-1-carboxamide (PCN) biosynthesis without inhibition in feedback by PCN production. It highlighted the importance of PhzC and applying *P*. *chlororaphis* for shikimate pathway-derived high-value biological production.

**Abstract:**

DAHP synthase catalyzes the first step in the shikimate pathway, deriving the biosynthesis of aromatic amino acids (Trp, Phe and Tyr), phenazine-1-carboxamide, folic acid, and ubiquinone in *Pseudomonas chlororaphis*. In this study, we identified and characterized one DAHP synthase encoding gene *phzC*, which differs from the reported DAHP synthase encoding genes *aroF*, *aroG* and *aroH* in *E*. *coli*. PhzC accounts for approximately 90% of the total DAHP synthase activities in *P*. *chlororaphis* HT66 and plays the most critical role in four DAHP synthases in the shikimate pathway. Inactivation of *phzC* resulted in the reduction of PCN production by more than 90%, while the absence of genes *aroF*, *aroG* and *aroH* reduced PCN yield by less than 15%, and the production of PCN was restored after the complementation of gene *phzC*. Moreover, the results showed that *phzC* in *P*. *chlororaphis* HT66 is not sensitive to feedback inhibition. This study demonstrated that gene *phzC* is essential for PCN biosynthesis. The expression level of both *phzC* and *phzE* genes are not inhibited in feedback by PCN production due to the absence of a loop region required for allosteric control reaction. This study highlighted the importance of PhzC and applying *P*. *chlororaphis* for shikimate pathway-derived high-value biological production.

## 1. Introduction

**Figure 1:**
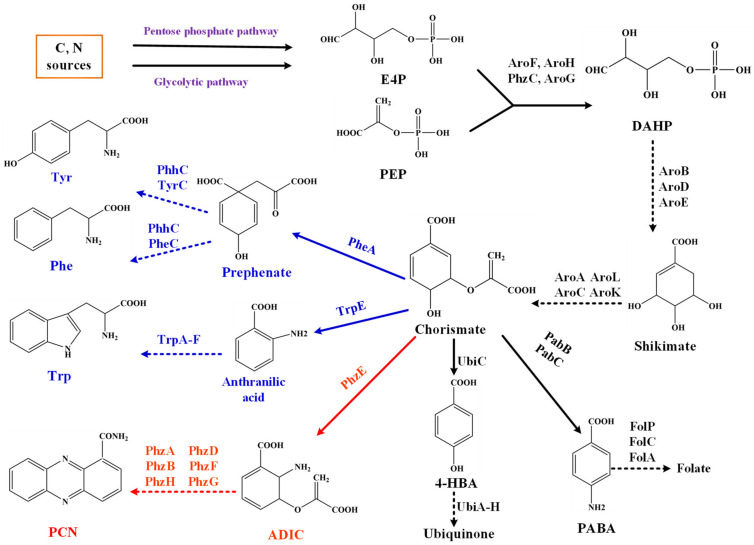
The shikimate pathway and chorismate-derived pathway in *P*. *chlororaphis* HT66.

The shikimate pathway is one of the most studied pathways in *E*. *coli*. Glucose and other carbon sources produce phosphoenolpyruvate (PEP) and erythrose-4-phosphate (E4P) through the glycolytic pathway (EPP) and pentose phosphate pathway (PPP), respectively. Both intermediates react to form 3-deoxy-arabino heptanoic acid-7-phosphate (DAHP) by the enzymatic action of DAHP synthase and enter the shikimate pathway to synthesize chorismate, which then further synthesizes an extensive range of valuable products through different metabolic routes [1,2]. Eight core enzymes, AroA, AroB, AroC, AroD, AroE, AroF (or isoenzymes AroG, AroH), AroL and AroK are involved in the shikimate pathway (Figure 1), and their biosynthetic mechanisms have already been explored [3,4]. It is the most common metabolic pathway for the biosynthesis of aromatic amino acids in plants, bacteria, and fungi. Microorganisms and plants use the shikimate pathway to synthesize aromatic amino acids and other important metabolites, including salicylic acid, folic acid, vitamin K, iron carrier, coenzyme Q and phenazine compounds [4,5,6]. Moreover, the aromatic amino acids, 4-hydroxybenzoic acid (4-HBA), gentisate, and other intermediates of the shikimate pathway are further derivatized in various metabolic pathways, resulting in the synthesis of more metabolites [7].

PEP and E4P combine to produce DAHP through a condensation reaction catalyzed by 3-deoxy-arabino heptulose-7-phosphate synthase. The engineered strain expressing an additional copy of the *dahp* gene showed enhanced glycopeptide production by approximately a factor of three. Deletion of *dahp* resulted in significant reduction in balhimycin production. Thus, the synthesis of DAHP is a critical step in determining the yield of compounds produced by the shikimate pathway [8].

The shikimate pathway is the leading pathway for the synthesis of numerous aromatic compounds and phenazine antibiotics. The genetically engineered strains for the shikimate pathway have mainly been reported to be *Escherichia coli* [9], *Bacillus subtilis* [10], *Corynebacterium glutamicum* [11], *Pseudomonas* [7,12] and yeast [13].

The production of shikimate pathway derivatives can be enhanced by increasing precursor supply, blocking the competitive routes and relieving negative regulation. To alleviate the stress of feedback inhibition, feedback suppression mutations have been used to enhance the yield of the target compounds. Overexpression of the feedback-suppressor mutant *aroG^FBR^* in *C*. *glutamicum* has surged the potential of shikimate pathway and increased the yield of 4-HBA by 21.2-fold [14]. In addition, *E. coli* DAHP synthases are mainly encoded by *aroF* and *aroG*, and overexpressing their respective feedback-suppressor mutant *aroG^FBR^, aroF^FFBR^* nullifies the effect of feedback inhibition and significantly promotes the yield of shikimate pathway-derived compounds, such as tryptophan, muconic acid [7,15], arbutin [16], maleate [6,17], 2-pyran-4, 6-dicarboxylic acid [18], phenazine-1-carboxylate acid, etc. [19]

Various studies have focused on using *E*. *coli* to produce the shikimate pathway products and aromatic amino acids. It has been identified that *Pseudomonas* spp. synthesizes phenazine compounds through the shikimate pathway (Figure 1). The first systematic analysis of the core phenazine biosynthetic enzymes in *Pseudomonas* was presented by Floss and coworkers [20]. The sequence analysis of phenazine biosynthetic gene cluster (phz-operon) from *P*. *fluorescens* 2–79 showed that gene *phzC* (one of the seven core enzymes involved in phenazine biosynthesis) is not conserved in all phenazine-producing strains. The gene *phzC* is homologous to three isozymes *aroF*, *aroG* and *aroH*. As mentioned earlier, studies explored that the DAHP synthases are sensitive to feedback-inhibition, but a complete sequence analysis of gene *phzC* indicated that the enzyme lacks a loop region required for allosteric control function and feedback inhibition [21]. The *phzC* gene is essential for producing phenazine-1-carboxylic acid (PCA) and generates chorismate by converting carbon metabolites into the shikimate pathway and ensuring sufficient precursor generation [7].

The amino acid sequence analysis of proteins PhzC from *P. fluorescens* and *P. aureofaciens* and the DAHP synthases from *Streptomyces coelicolor* and *S. rimosus* revealed that these are typical type II enzymes [22]. Since the sequence of DAHP synthases from plants shows a high degree of homology with the indicated microbial DAHP synthases [23,24], plant DAHP synthases differ from bacterial DAHP synthases and are not sensitive to feedback inhibition [25]. Here, neither the sequence of protein nor the location of the gene *phzC* provides insights into the derivation of the phenazine nucleus [26,27].

*Pseudomonas chlororaphis* HT66 is a plant growth-promoting rhizobacterium (PGPR) capable of producing PCN with four genes encoding DAHP synthase. This study aims to uncover the essentiality of the DAHP synthase encoding gene *phzC* for the shikimate pathway and biosynthesis of phenazine-1-carboxamide (PCN). *Pseudomonas chlororaphis* HT66 has been studied for many years in our laboratory to synthesize phenazine compounds, such as PCA, PCN, 2-hydroxyphenazine, and 2,3-dihydro-3-hydroxyanthranilic acid (DHHA). These compounds were obtained through metabolic engineering and gene manipulations based on the shikimate pathway [28].

E4P, erythrose-4-phosphate; PEP, phosphoenolpyruvate; DAHP, 3-deoxy-Darabino-heptulosonate7-phosphate; PCN: phenazine-1-carboxamide; 4-HBA, 4-hydroxybenzoic acid; Tyr: tyrosine; Trp: Tryptophan; Phe: Phenylalanine. Main enzymes involved: AroF, AroH, PhzC, AroG: DAHP synthase; PhzE: anthranilate synthase. The phenazine biosynthesis was marked in red, and the aromatic amino acid biosynthesis was marked in blue.

*P*. *chlororaphis* HT66 can efficiently synthesize phenazine compound PCN, the gene *phzC* encodes DAHP synthase and is mainly involved in condensing E4P and PEP to form DAHP in *P*. *chlororaphis* HT66 (Figure 1). To explore the function of PhzC in the biosynthesis of shikimate pathway derivatives, we explained the effect of gene *phzC* on PCN biosynthesis by *P*. *chlororaphis* HT66. The analysis of gene *phzC* function will lay the foundation for the cell factory construction in *Pseudomonas* based on the shikimate pathway.

## 2. Materials and Methods

### 2.1. Bacterial Strains, Plasmids, and Growth Conditions

All strains used and engineered in this study are listed in Table 1, and the oligonucleotides are summarized in Table 2. Luria-Bertani (LB) medium (Tryptone 10 g, Yeast extract 5 g, NaCl 10 g, g/L) was used to incubate *E.* coli and *P. chlororaphis* during the mutant’s construction. *E. coli* was incubated at 37 °C, while *P. chlororaphis* was cultured at 28 °C. King’s medium B (KB) medium (Glycerol 18 g, Tryptone 20 g, MgSO_4_ 0.732 g, K_2_HPO_4_ 0.514 g, g/L), K medium (Glycerol 18 g, MgSO_4_ 0.732 g, K_2_HPO_4_ 0.514 g, g/L) and G medium (Tryptone 20 g, MgSO_4_ 0.732 g, K_2_HPO_4_ 0.514 g, g/L) were used for activating *P. chlororaphis* for fermentation. Precisely, 100 μg mL^−1^ of ampicillin and 50 μg mL^−1^ of kanamycin were added to the medium for selection.

For shake-flask fermentation, *P*. *chlororaphis* and its derivatives were activated at 28 °C overnight in agar media. A single colony was inoculated in 60 mL flasks for 12 h at 28 °C with 200 rpm shaking, and then 0.6 mL of the culture was inoculated for fermentation in 60 mL KB medium for 64 h at 28 °C with 200 rpm.

### 2.2. DNA Manipulation and Transformation

All plasmids used and constructed in this study are listed in Table 1. All genes were amplified with PrimerSTAR Max DNA Polymerase (Takara Bio., Beijing, China). Plasmids containing respective genes were constructed with In-Fusion Cloning Kit (Takara Bio.) Chromosomal in-frame deletions of *phzC* were performed separately using the method previously reported [7].

Plasmid pBBR1-MCS2 was used to construct the *phzC* overexpression strain using native phenazine promoter P*_phz_*. First, gene *phzC* and P*_phz_* promoter from the genomic DNA of *P*. *chlororaphis* HT66 were amplified. Then, gene *phzC* and promoter P*_phz_* fragments were simultaneously inserted into the plasmid pBBR1-MCS2 using an In-Fusion HD Cloning Kit (Takara Bio.) yielding the plasmid pBBR-P*_phz_*-*phzC*. The plasmid was verified through PCR and DNA sequencing before transforming into *P*. *chlororaphis* by electroporation (Bio-Rad, Hercules, CA, USA). All of the primers used are shown in Table 2.

### 2.3. Sequence and Protein Analysis of DAHP

DNA sequences of the genes in *P*. *chlororaphis* were retrieved from the *Pseudomonas* Genome Database (http://www.pseudomonas.com/, accessed on 6 December 2019). Sequence homology searching was conducted using the NCBI nucleotide BLAST server. The amino acid sequences of DAHP synthase from other strains were obtained from GenBank. A phylogenetic tree was constructed by MEGA 7.0 using the Neighbor-Joining method.

First, the SWISS-MODEL template library (SMTL version 2020-08-05, PDB release 2020-07-31) [29,30] was searched with BLAST [31] and HHBlits [32] for evolutionary related structures matching the sequence of PhzC. Then, the obtained model was prepared using AutoDock Tools (ADT) [33] and converted into pdbqt mode. Through the site finder function of Molecular Operating Environment (MOE), we predicted a binding site, which coincides the positions and reactions mechanism of the two ligands observed in previous studies [34]. Finally, we used MOE to create dummy atoms as the center of the box, and then calculated the affinity of the PhzC with PEP and EFP.

### 2.4. Fermentation Process of P. chlororaphis and its Derivative Strains

*P*. *chlororaphis* and its derivatives strains were activated overnight at 28 °C in KB agar media. Selection of single colonies from Petri plates was performed and used to inoculate approximately 5 mL of KB broth in 50 mL flasks. The primary cultures were incubated overnight at 28 °C at 200 rpm. Portions of these cultures were inoculated into 250 mL baffled flasks containing 60 mL KB broth to achieve an initial OD_600_ of 0.02. The fermentation process was initiated, and the samples were collected every 12 h to determine cell growth and PCN concentration. When genes were overexpressed in vector pBBR1-MCS2, after 6 h of cultivation, isopropyl *β*-D-1-thiogalactopyranoside (IPTG) was added to the culture at a final concentration of 0.1 mM. Triplicate experiments were carried out for each fermentation test.

### 2.5. Quantitative Real-Time PCR

*P*. *chlororaphis* and its derivatives were cultured in KB medium for 26 h, RNA preparation was performed using TaKaRa MiniBEST Universal RNA Extraction Kit. RNA stock was prepared as reported by harvesting cells [7]. Total RNA (500 ng) was immediately used in reverse transcription using the PrimeScript™ RT reagent Kit. The qPCR reactions were performed in a 96-well plate with a 10 µL mixture containing 5 µL 2 × SYBR green^®^ Premix DimerEraser™ (Takara), 1 µL ROX reference dye, 1 µL cDNA, and appropriate primer concentration. All samples were analyzed in triplicate. The relative expression levels were calculated based on the reported methods [35]. The *rpoD* gene encoding sigma factor was used as a reference. The fold change for mRNA was calculated by the 2^−ΔΔCt^ method. The gene modules were identified using the methods of Fragments Per Kilobase Million (FPKM) [total_exon_fragments/mapped reads (millions) exon length (kB)] [36]. Calculation of the copy numbers of target genes was supplied in supporting information.

### 2.6. Quantitative Assay for PCN Production

To extract PCN, 500 μL of fermentation culture was mixed with 25 μL of 6 M HCl, and then 3500 μL of ethyl acetate was used for extraction. PCN was separated using HPLC (Agilent Technologies 1260 Infinity, Santa Clara, CA, USA) with a C18 reversed-phase column (5 μm, 4.6 × 12.5 mm) eluted with acetonitrile-5 mM ammonium acetate (60:40, *v*/*v*). PCN production was quantified using peak area (A) in HPLC elute according to the following formula: PCN (mg/L) = 0.00871A − 3.6617, which was derived from a dose-peak area plot using purified PCN with a correlation coefficiency (R^2^) of 0.999. Dry cell weight (DCW) in KB medium was calculated from the optical density at 600 nm (1OD_600_ = 0.4135 g DCW L^−1^).

### 2.7. Statistical Analysis

The results were averaged and presented as the mean ± standard deviation from triplicate independent experiments. Significant differences between means (*p* < 0.05) were determined by one-way analysis of variance followed by Duncan’s multiple range test (SAS Institute Inc., Cary, NC, USA).

## 3. Results

### 3.1. Uncovering DAHP Synthases in P. chlororaphis HT66

**Figure 2:**
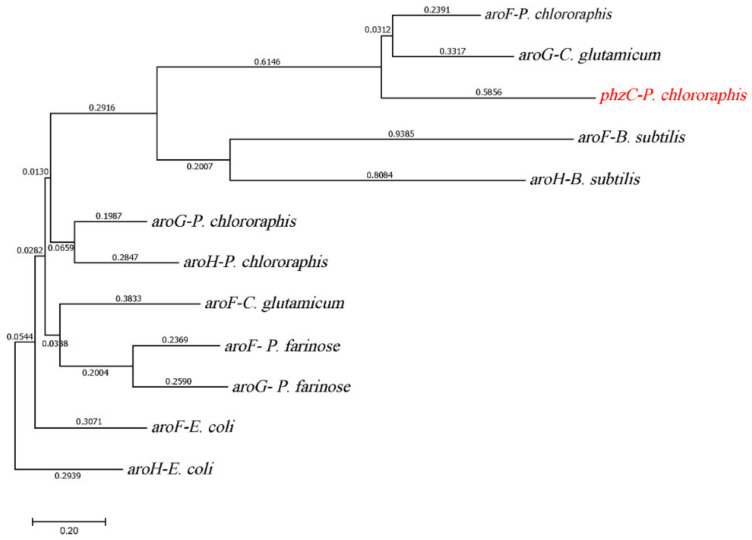
Phylogenetic tree constructed based on the alignment of DAHP synthases.

The initial step of the shikimate pathway is the synthesis of DAHP from the precursor’s PEP and E4P catalyzed by DAHP synthase (EC 2.5.1.54) [37]. The gene *aroG* sequence was used against the *P*. *chlororaphis* genome in BLAST search analysis. Interestingly, four genes encoding DAHP synthases were found in *P*. *chlororaphis,* whereas three DAHP synthase isozymes are harbored in *E*. *coli*. One DAHP synthase encoded by gene *phzC* was only found in *Pseudomonas*. Phylogenetic analysis was carried out to determine the differences between DAHP synthases based on amino acid sequence alignment in *P*. *chlororaphis* and *E*. *coli*. Figure 2 showed that two types of DAHP synthases were identified, four genes encoding DAHP synthase in *P*. *chlororaphis* HT66 genomic database are M217_RS0108615 (*aroF*), M217_RS0108710 (*aroG*), M217_RS0131975 (*aroH*) and M217_RS0112890 (*phzC*). The amino acid sequence of PhzC in *P*. *chlororaphis* HT66 is 24%, 40% and 36%, identical to the amino acid sequence of AroF, AroG and AroH of *E*. *coli*, respectively. The AroF of *P*. *chlororaphis* HT66 is homologous to AroF, AroG and AroH in *E*. *coli* with the amino acid sequence at 26%, 29% and 36%, respectively. Moreover, AroG amino acid sequence is 60%, 54% and 47% identical to the amino acid sequences of AroF, AroG and AroH in *E*. *coli*, respectively. Similarly, AroH amino acid sequence is 53%, 49% and 46% identical to AroF, AroG and AroH in *E*. *coli*, respectively. Sequence analysis of different DAHP synthases showed that DAHP synthases were classified into DAHP synthase type I and DAHP synthase type II, and PhzC from *P*. *chlororaphis* HT66 belongs to DAHP synthase type II. Comparative analysis of PhzC in phenazine producing and non-PHZ producing strains was shown in Table S1 in Supplementary File.

**Figure 3:**
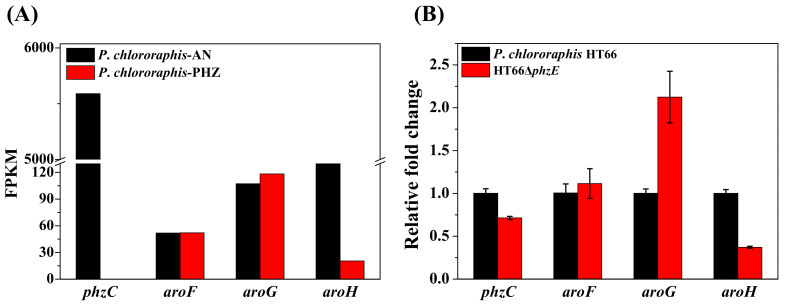
The expression differences of different DAHP synthase enzymes by transcriptome and relative quantitative PCR analysis. (**A**) Based on transcriptome analysis; (**B**) Based on relative quantitative PCR analysis.

*phzC* encodes the first DAHP synthase in the shikimate pathway, *aroF*, *aroG* and *aroH* encode the next three DAHP synthases in *P*. *chlororaphis,* whereas only *aroF*, *aroG* and *aroH* are found in *E*. *coli*. Based on our earlier transcriptomic analysis, the FPKM (fragments per kilobase of exon per million fragments mapped) of four DAHP synthases were shown in Figure 3. As illustrated, the FPKM value of gene *phzC* was 5590 in the 2-hydroxyphenazine-accumulating strain *P*. *chlororaphis*-AN, which was significantly higher than *aroF* (FPKM = 107), *aroG* (FPKM = 52) and *aroH* (FPKM = 898). FPKM values are 108 times, 52 times, and 6 times of that genes *aroF*, *aroG* and *aroH*, respectively, and it was similar to the previous iTRAQ-based quantitative proteomic analysis [38]. Based on the transcriptomic analysis of phenazine-deficient strain *P*. *chlororaphis*-PHZ, no significant change was observed in the transcriptional levels of *aroF* (FPKM = 117), *aroG* (FPKM = 52), and *aroH* (FPKM = 20). Thus, *phzC* is hypothesized to be more essential for the biosynthesis of phenazine derivatives than *aroF*, *aroG*, and *aroH* in *P*. *chlororaphis* HT66.

The *P*. *chlororaphis*-AN (with strengthened phenazine synthetic pathway) and *P*. *chlororaphis*-AN-PHZ (with inactivated phenazine synthetic pathway) were used for transcriptome analysis.

For further analysis, the PCN biosynthetic pathway was blocked in *P*. *chlororaphis* HT66, resulting in phenazine-deficient strain HT66Δ*phzE*. After 26 h of incubation, the transcription level of different DAHP synthases was noted. Figure 3B showed that gene *phzC* is down-regulated by 1.4-fold, *aroF* and *aroG* are up-regulated by 1.1-fold, 2.1-fold respectively, while gene *aroH* is down-regulated by 2.7-fold after inactivating phenazine synthetic pathway. The transcription level is consistent with both inactivated strains of the entire phenazine synthase gene cluster (*P*. *chlororaphis*-PHZ) and phenazine biosynthetic pathway (HT66Δ*phzE*) (Figure 3). The transcriptional level of *phzC* was 29% decreased after deleting *phzE* gene. Thus, it was supposed that changes in the transcriptional levels of genes *aroF*, *aroG* and *aroH* are caused by the inactivation of *phzC* gene, which has a significant influence on the carbon flux of the entire shikimate pathway, and the resulting end products are responsible for the transcriptional regulation of *aroF*, *aroG* and *aroH*. Therefore, it was concluded that *phzC* is essential for PCN biosynthesis.

### 3.2. Mutation and Functional Characterization of DAHP Synthases

**Figure 4:**
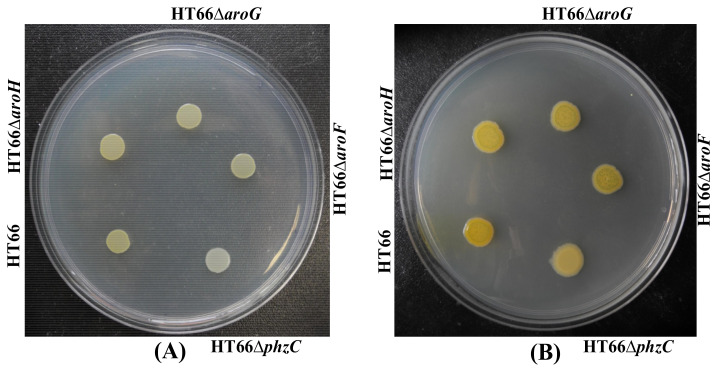
Photographs of the different derivatives grown on KB medium. (**A**) 24 h; (**B**) 48 h.

**Figure 5:**
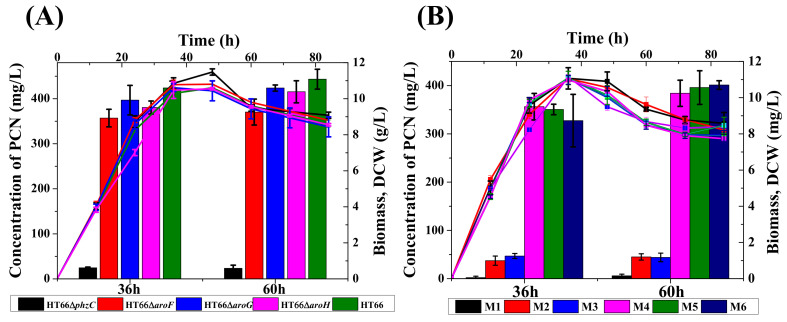
Characteristics growth profile and PCN synthesis of different DAHP synthase-deficient strains. (**A**) Single-deletion, (**B**) Dual deletion. M1: HT66Δ*phzC*Δ*aroF*, M2: HT66Δ*phzC*Δ*aroG*, M3: HT66Δ*phzC*Δ*aroH*, M4: HT66Δ*aroF*Δ*aroG*, M5: HT66Δ*aroF*Δ*aroH*, M6: HT66Δ*aroG*Δ*aroH*. DCW (line), Concentration of PCN (column).

To explore the function of four different DAHP synthases in *P*. *chlororaphis*, single DAHP synthase defective strain was constructed through non-scar gene deletion [7]. The derived strains were cultured in KB medium for 72 h. As shown in Figure 4, no morphological difference was found, whereas *phzC* mutant strain HT66Δ*phzC* displayed a reduction in PCN production. The cell growth and PCN titer were checked in KB medium after 84 h of incubation to further analyze PCN production. Figure 5A showed no significant differences in the cell growth of mutant strains of genes *phzC*, *aroF*, *aroG* and *aroH*. Surprisingly, the production of PCN was dramatically decreased by the inactivation *phzC* gene, but the effect of *aroF*, *aroG* and *aroH* genes was less efficient on PCN production (Figure 5A). The production of PCN was 24.6 mg/L, 357.1 mg/L, 396.8 mg/L and 380.5 mg/L on the single deletion of *phzC*, *aroF*, *aroG* and *aroH* respectively. After 36 h of cultivation, four mutants produced 5.8%, 84.2%, 96.6%, and 89.7% of the PCN concentration compared with the wild-type strain, respectively. Consequently, the PCN production was significantly reduced by knocking out *phzC*. No significant titer of aromatic amino acids was detected (data not shown).

To further investigate the effect of *phzC* on PCN production, double DAHP synthases genes were knocked out by constructing six individual mutant strains. Both the cell growth and PCN production were assessed in KB medium, as shown in Figure 5B. Results indicated no significant difference in cell growth among all the derivatives strains. HPLC analysis showed that the PCN production was dramatically decreased to 2.3 mg/L, 37.3 mg/L and 46.9 mg/L in the mutant strain HT66Δ*phzC*Δ*aroF*, HT66Δ*phzC*Δ*aroG* and HT66Δ*phzC*Δ*aroH*, compared to 0.54%, 8.8%, and 9.93% of the PCN produced by the wild-type strain, respectively. The inactivation of gene *phzC* showed that *phzC* is essential for PCN biosynthesis. However, the PCN production was decreased by 84%, 82.5%, and 77.1% in the double mutant strains HT66Δ*aroF*Δ*aroG*, HT66Δ*aroF*Δ*aroH*, HT66Δ*aroG*Δ*aroH*, respectively. These results were consistent with the mutant strains with *aroF*, *aroG* and *aroH* knockout, which showed less significant function in PCN biosynthesis, as revealed by a single gene DAHP synthase inactivation. In the absence of *phzC*, PCN production was decreased by more than 90%, while the inactivation of genes *aroF*, *aroG* and *aroH* had a 15% or less significant effect on PCN production. In addition, the loss of *phzC* reduces the flow of carbon fluxes in the shikimate pathway leading to a substantial reduction in PCN production in *P*. *chlororaphis*.

### 3.3. Gene Expression Level and Its Quantification

**Figure 6:**
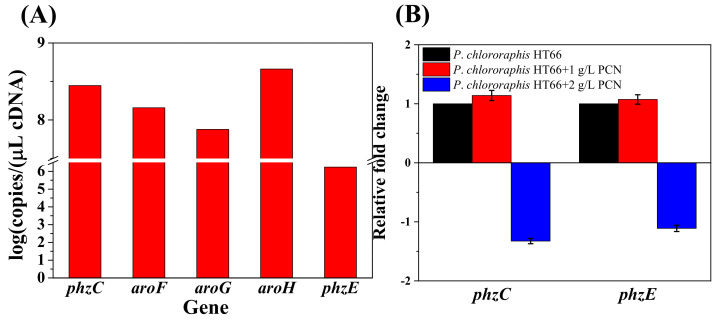
Transcriptional level of different DAHP synthase genes and inhibition of PCN on *phzC* and *phzE* transcription. (**A**) Based on absolute quantification; (**B**) Based on relative quantification. Calculation of the copy numbers of target gene was shown in Table S4 in Supplementary File.

To better understand the difference among the AroF, AroG, AroH and PhzC in PCN biosynthesis, the transcriptional level of DAHP synthases encoding genes was measured after culturing for 26 h. As shown in Figure 6A, the absolute transcriptional level of *phzC* was normalized to 8.4, higher than *aroF* (8.2) and *aroG* (7.9), lower than *aroH* (8.7), so no significant expression difference was found in the derived strains of *P*. *chlororaphis* HT66 (Tables S2 and S3 in Supplementary File). It reveals that gene *phzC* is essential for PCN biosynthesis, which is not dependent on its higher expression level. Moreover, PhzE is the first enzyme that catalyzes chorismate for phenazine derivative synthesis. We also determined the absolute transcript level of gene *phzE* in the wild-type strain and found that the transcription level of *phzC* was significantly higher than *phzE* (Figure 6A). It was supposed that *phzC* predominantly determines the efficiency of the shikimate pathway, and its high transcriptional level may be involved in the shikimate pathway productivity. 

As the previous studies reported, the transcriptional level of *aroF*, *aroG* and *aroH* were inhibited by aromatic amino acids in *E*. *coli*. In this study, the feedback inhibition of genes *phzC* and *phzE* was studied in strain *P. chlororaphis* HT66. Strain *P. chlororaphis* HT66 was cultured in KB medium for 12 h, and PCN was added to the medium at a final concentration of 1 g/L and 2 g/L, respectively. After 26 h of culturing, the transcriptional levels of genes *phzC* and *phzE* were determined. After adding different concentrations of PCN, the transcription level of *phzC* was up-regulated by 1.14 times (PCN, 1 g/L) and down-regulated by 1.32 times (PCN, 2 g/L), and no significant feedback inhibition was observed (Figure 6B). Therefore, it is speculated that PhzC and PhzE in *P*. *chlororaphis* HT66 were not sensitive to feedback inhibition by PCN (pathway end-product).

### 3.4. Effect of DAHP Synthase Gene Complementation in the Mutant Strains

**Figure 7:**
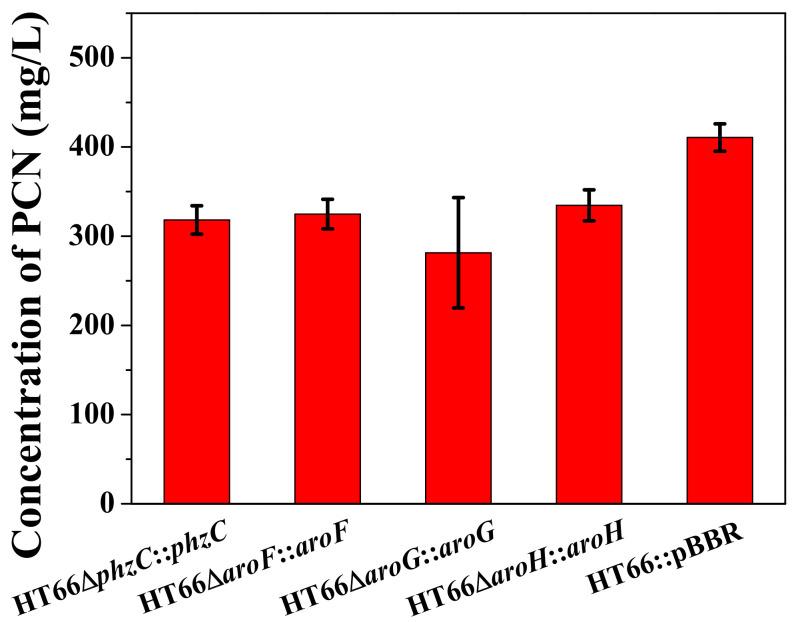
The production of PCN in different DAHP synthase overexpression strains.

Based on the pBBR1MCS-2 plasmid and the previously screened native P*_phz_* promoter, *phzC*, *aroF*, *aroG* and *aroH* were overexpressed and restored into the corresponding single DAHP synthase deficient strain, and the empty plasmid was expressed in the wild type strain as a control, HT66Δ*phzC*::*phzC*, HT66Δ*aroF*::*aroF*, HT66Δ*aroG*::*aroG*, HT66Δ*aroH*::*aroH* and HT66::pBBR were constructed respectively. The fermentation process was carried out in the shake flask containing KB medium, and PCN production was measured after 36 h (Figure 7). PCN production was restored to 318.2 mg/L when *phzC* was overexpressed in the *phzC* defective strain, which was 78% and 74.9% compared with HT66::pBBR and wild type strain, respectively. It is also shown that expressing an empty plasmid pBBR-MCS2 in *P*. *chlororaphis* HT66 had a slight effect on PCN production, while our previous study reported that antibiotics and inducers displayed a notable effect on the growth of *P*. *chlororaphis* [7].

### 3.5. Effect of DAHP Synthases on the Metabolism of Carbon and Nitrogen Sources

**Figure 8:**
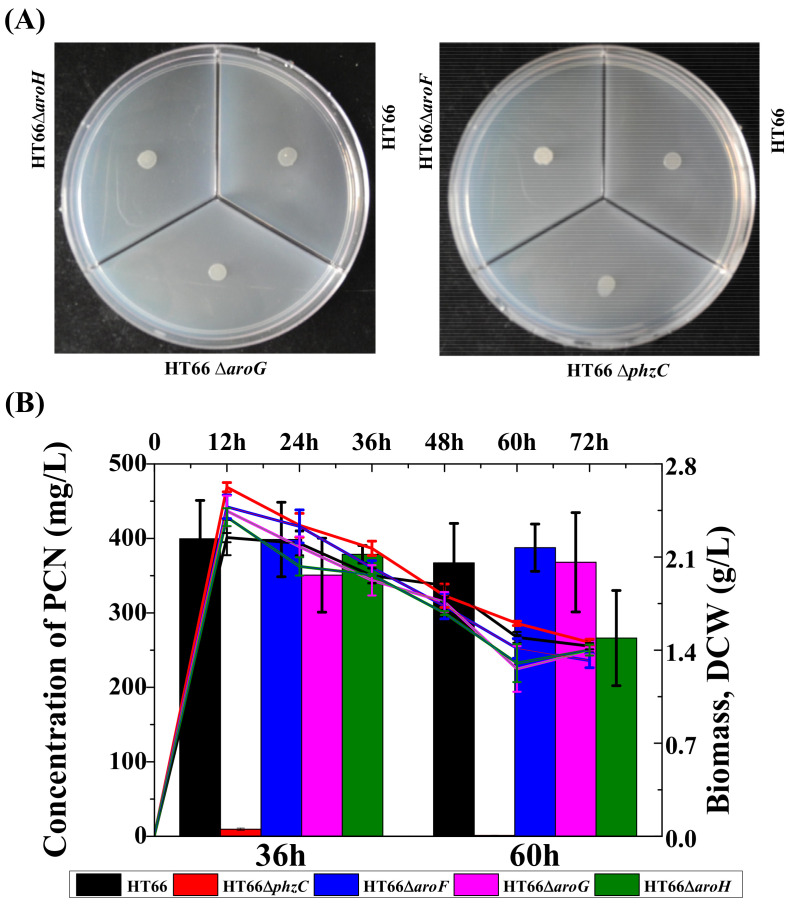
Photographs of *P*. *chlororaphis* HT66 derivatives grown on specific medium. (**A**) KB medium without tryptone, (**B**) KB medium without glycerol.

PhzC catalyzes the condensation reaction of PEP and E4P to produce metabolite DAHP and is essential for PCN biosynthesis in *P*. *chlororaphis* HT66. To investigate the effect of DAHP synthases on the carbon metabolism in *P*. *chlororaphis*, strains HT66Δ*phzC*, HT66Δ*aroF*, HT66Δ*aroG* and HT66Δ*aroH* were incubated in a medium (MM+glycerol) containing glycerol as the sole carbon source. Results showed no significant difference in colony morphology and the cell growth among different DAHP synthase-deficient strains (Figure 8A). It is indicated that the absence of different DAHP synthase does not affect glycerol utilization.

As shown in Figure 8B, when the strains HT66, HT66Δ*phzC*, HT66Δ*aroF*, HT66Δ*aroG* and HT66Δ*aroH* were grown in glycerol deficient medium, no significant reduction in PCN production was observed, but the absence of glycerol in the medium reduced the maximum biomass significantly (OD_600_ only reached 6.4). After culturing for 36 h, the concentration of PCN produced by HT66Δ*phzC*, HT66Δ*aroF*, HT66Δ*aroG*, and HT66Δ*aroH* was 9.5 mg/L, 398.5 mg/L, 350.5 mg/L and 378.7 mg/L, respectively. No significant difference for PCN production was observed in the KB medium. Moreover, *P*. *chlororaphis* HT66 could produce 399.5 mg/L of PCN without glycerol, 10% less than that harvested in KB medium. The results indicated that gene *phzC* displayed no effect on the metabolism of carbon and nitrogen sources. Thus, combining with our early study, it is found that *P*. *chlororaphis* HT66 utilizes tryptone as a priority nutrient and then enters glycerol metabolism.

## 4. Discussion

Recently, DAHP synthases were grouped into two distinct classes based on protein sequence homology. The NCBI blasting analysis of DAHP synthase encoding genes shows that, in addition to genes *aroF*, *aroG* and *aroH*, *P*. *chlororaphis* HT66 also harbor another gene *phzC* encoding DAHP synthase. In *E*. *coli*, AroG accounts for approximately 80% of the total DAHP synthase activity exhibiting a higher specific proteolytic resistance than other DAHP synthases [39,40]. However, *phzC* was deemed about 90% of the total DAHP synthase activity for PCN synthesis in *P*. *chlororaphis* HT66. Once *phzC* was inactivated, the production of PCN decreased by more than 90%, indicating that the PhzC is mainly responsible for DAHP synthase activity.

**Figure 9:**
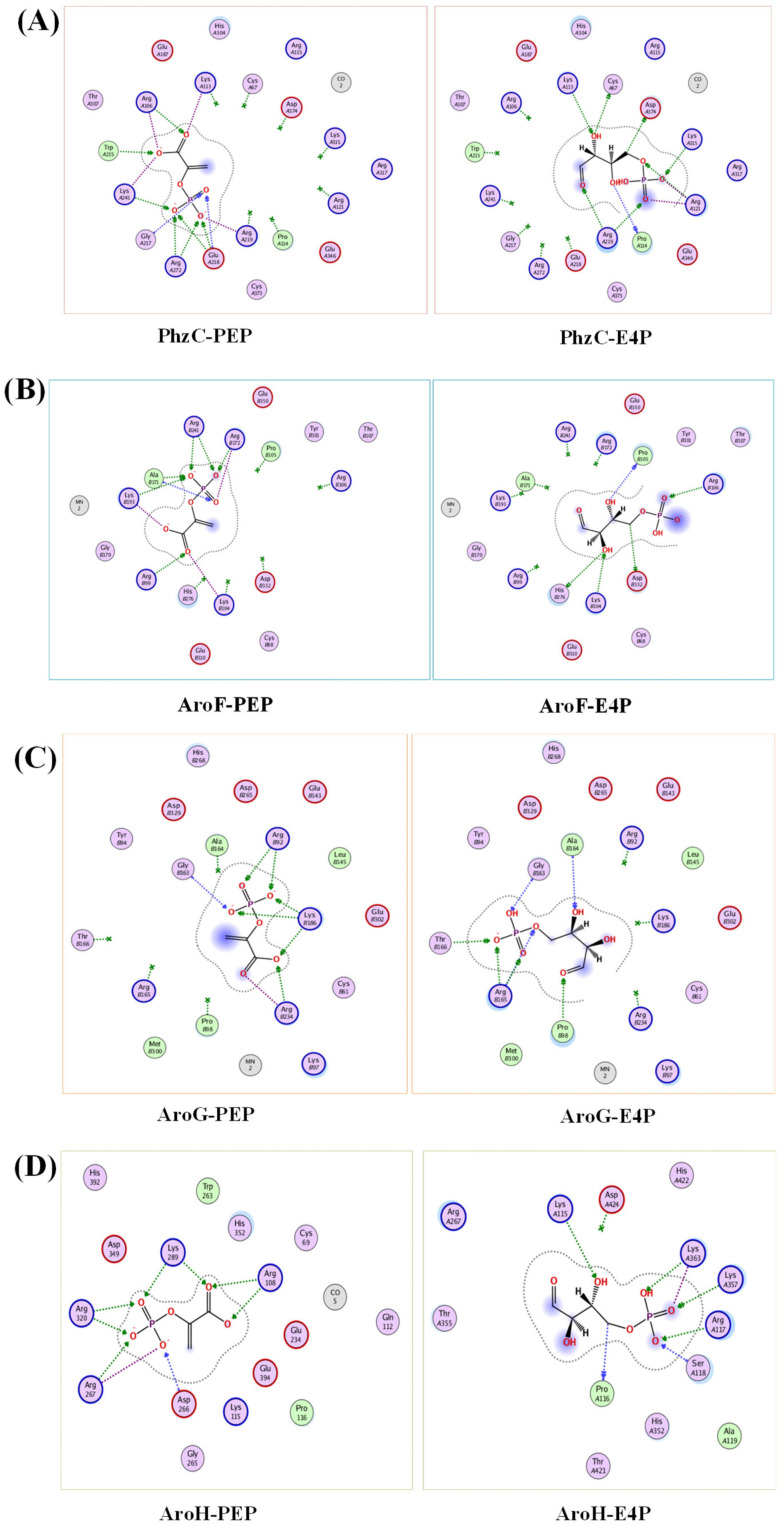
Molecular docking of DAHP synthases in *P*. *chlororaphis*. Analysis of the binding mode of PEP and E4P with PhzC (**A**), AroF (**B**), AroG (**C**), AroH (**D**).

In addition, *aroF, aroG* and *aroH* have been widely studied in *E*. *coli* and are sensitive to feedback inhibition to aromatic amino acids, tyrosine, phenylalanine and tryptophan, respectively [41,42,43]. In contrast, by adding different concentrations of PCN to the culture medium, it was found that gene *phzC* was not sensitive to feedback inhibition to the pathway end products PCN. Moreover, no feedback inhibition was observed when feeding aromatic amino acids (data not shown). The previous studies have found that *phzC* in *P*. *fluorescens* 2–79 is not conserved in different phenazine producing strains. Unlike *aroF*, *aroG* and *aroH*, *phzC* lacks an allosteric control loop region and is not sensitive to metabolites feedback inhibition [21]. When using the swiss-model to perform homology modeling, as seen in Figure 9, the affinity of PhzC with PEP and E4P are −5.9 kcal/mol and −5.4 kcal/mol, respectively. The affinity of AroF with PEP and E4P are −6.6 kcal/mol and −5.7 kcal/mol, respectively. The affinity of AroG with PEP and E4P are −5.2 kcal/mol and −4.6 kcal/mol, respectively. The affinity of AroH with PEP and E4P are −6.6 kcal/mol and −5.9 kcal/mol, respectively (Figure 9).

DAHP synthase catalyzes the condensation of E4P and PEP to produce DAHP, which is a key step in determining the yield and production of shikimate pathway-based derivatives. Our study found that the loss of different DAHP synthase in *P*. *chlororaphis* HT66 did not affect the utilization of carbon sources, such as glycerol. Furthermore, chorismate is converted to ADIC by the biocatalysis of PhzE, and introduces a nitrogen atom through glutamate amino acid, similar to the anthranilate synthase [44]. The PhzF catalyzes the isomerization of DHHA by obtaining a proton from the C3 position of DHHA using a molecule of glutamic acid. In addition, PhzB promotes the formation of diimine hexahydro-phenazine-1,6-dicarboxylic acid (HHPDC) by a two-step catalytic reaction, which is catalyzed by glutamic acid and a pair of histidine/serine, respectively [45]. Amino acids play an essential role in the synthesis of phenazine compounds. When glycerol or tryptone was used as the sole carbon or nitrogen source, different DAHP synthase deficient strains utilized glycerol and tryptone actively. The loss of DAHP synthases did not affect the utilization of carbon and nitrogen sources in *P*. *chlororaphis* HT66. At the same time, only tryptone acted as the sole energy source in wild-type strain HT66 resulting in satisfactory production of PCN. In the case of P3 (Derivative strain of *P*. *chlororaphis* HT66) strain [46], glycerol is the main carbon source responsible for the high yield of PCN. The above results indicated that the carbon and nitrogen-fed fermentation could further increase the yield of the shikimate pathway compound.

It has been revealed that the dominant role of gene *phzC* in the biosynthesis of PCN in *P*. *chlororaphis* HT66 was not from the difference in transcription levels, but it can be assumed that *phzC* mainly determines the carbon flux of the shikimate pathway and its expression is not sensitive to feedback inhibition to the pathway metabolites. These results will promote the construction of the *P*. *chlororaphis* HT66 cell factory based on the derivatives of the shikimate pathway.

## 5. Conclusions

In this study, we identified and characterized DAHP synthase encoding gene *phzC*, which differs from the reported DAHP synthases *aroF*, *aroG* and *aroH* in *E*. *coli*. It was proved that PhzC is essential for PCN biosynthesis in the strain *P*. *chlororaphis* HT66. Inactivation of gene *phzC* resulted in the reduction of PCN yield by more than 90%, while the absence of genes *aroF*, *aroG* and *aroH* reduced PCN yield by less than 15%. After the complementation of gene *phzC* in the corresponding DAHP synthase-deficient strain, the production of PCN was significantly restored. PhzC has a significant effect on PCN biosynthesis, and its role on the carbon flux of the shikimate pathway in *P*. *chlororaphis* HT66 is more robust than that of AroF, AroG and AroH. Moreover, the results showed that the gene *PhzC* in *P*. *chlororaphis* HT66 is not sensitive to feedback inhibition and proved that production of high value-added compounds based on the shikimate pathway is feasible and advantageous in *P. chlororaphis* HT66 cell factory.

## Figures and Tables

**Table 1:** Main strains, plasmids used and developed in this study.

Strains	Description	Source
S17-1 (λ pir)	*E*. *coli* res^-^ pro mod^+^ integrated copy of RP4, mob^+^, used for incorporating constructs into *P*. *chlororaphis*	Lab stock
*P. chlororaphis* HT66	*P. chlororaphis* wild-type, PCN, Ap^r^, Sp^r^	Lab stock
HT66Δ*phzE*	*P. chlororaphis* HT66 with *phzE* deleted	This study
HT66Δ*phzC*	*P. chlororaphis* HT66 with *phzC* deleted	This study
HT66Δ*aroF*	*P. chlororaphis* HT66 with *aroF* deleted	This study
HT66Δ*aroG*	*P. chlororaphis* HT66 with *aroG* deleted	This study
HT66Δ*aroH*	*P. chlororaphis* HT66 with *aroH* deleted	This study
HT66Δ*phzC*Δ*aroF*	*P. chlororaphis* HT66 with *phzC*, *aroF* deleted	This study
HT66Δ*phzC*Δ*aroG*	*P. chlororaphis* HT66 with *phzC*, *aroG* deleted	This study
HT66Δ*phzC*Δ*aroH*	*P. chlororaphis* HT66 with *phzC*, *aroH* deleted	This study
HT66Δ*aroF*Δ*aroG*	*P. chlororaphis* HT66 with *aroF*, *aroG* deleted	This study
HT66Δ*aroH*Δ*aroG*	*P. chlororaphis* HT66 with *aroH*, *aroG* deleted	This study
HT66Δ*aroF*Δ*aroH*	*P. chlororaphis* HT66 with *aroF*, *aroH* deleted	This study
HT66::pBBR	*P. chlororaphis* HT66 harboring pBBR	This study
HT66::*phzC*	*P. chlororaphis* HT66 harboring pBBR-*P_phz_*-*phzC*	This study
HT66::*aroF*	*P. chlororaphis* HT66 harboring pBBR-*P_phz_*-*aroF*	This study
HT66::*aroG*	*P. chlororaphis* HT66 harboring pBBR-*P_phz_*-*aroG*	This study
HT66::*aroH*	*P. chlororaphis* HT66 harboring pBBR-*P_phz_*-*aroH*	This study
HT66Δ*phzC*::*phzC*	*P. chlororaphis* HT66Δ*phzC* harboring pBBR-*phzC*	This study
HT66Δ*aroF*::*aroF*	*P. chlororaphis* HT66Δ*aroF* harboring pBBR-*aroF*	This study
HT66Δ*aroG*::*aroG*	*P. chlororaphis* HT66Δ*aroG* harboring pBBR-*aroG*	This study
HT66Δ*aroH*::*aroH*	*P. chlororaphis* HT66Δ*aroH* harboring pBBR-*aroH*	This study
**Plasmids**	**Description**	**Source**
pk18*mobsacB*	Broad-host-range gene replacement vector, Km^r^	Lab stock
pk18-*phzE*	pk18*mobsacB* containing *phzE* upstream and *phzE* downstream, Km^r^	This study
pk18-*phzC*	pk18*mobsacB* containing *phzC* upstream and *phzC* downstream, Km^r^	This study
pk18-*aroF*	pk18*mobsacB* containing *aroF* upstream and *aroF* downstream, Km^r^	This study
pk18-*aroG*	pk18*mobsacB* containing *aroG* upstream and *aroG* downstream, Km^r^	This study
pk18-*aroH*	pk18*mobsacB* containing *aroH* upstream and *aroH* downstream, Km^r^	This study
pBBR-P*_phz_*-*phzC*	pBBR-MCS2 containing P*_phz_*-*phzC*, Km^r^	This study
pBBR-P*_phz_*-*aroF*	pBBR-MCS2containing P*_phz_*-*aroF*, Km^r^	This study
pBBR-P*_phz_*-*aroG*	pBBR-MCS2containing P*_phz_*-*aroG*, Km^r^	This study
pBBR-P*_phz_*-*aroH*	pBBR-MCS2containing P*_phz_*-*aroH*, Km^r^	This study

**Table 2:** Main primers designed and used in this study.

Primer	Sequence (5′–3′)
**For Gene Deletion**	
phzC-1F	CATGATTACGAATTCACAACTAACCGCTAGCGACACCACT
phzC-1R	GATGCGATCACTCTCACGAGAGAATT
phzC-2F	TGCGCTTGAACTCAGGAGTCTTTGCCTGGAGTTTGTCGCCATGACCG
phzC-2R	GACTCTAGAGGATCCGGTGGAAATCAGTACCCCGACATG
aroF-1F	CATGATTACGAATTCAGTTCGATGGCCTCGACGTCTTC
aroF-1R	CATGGACTCGGGTGTTTTTTAAGGT
aroF-2F	ACCTTAAAAAACACCCGAGTCCATGACCCGTAGCGCTCGATCATCC
aroF-2R	GACTCTAGAGGATCCGAAGCAAGCGGCCTATTGCCT
aroG-1F	CATGATTACGAATTCACGGTTGCACACTATCAGCCTCG
aroG-1R	CGTGTTACTCGTCAGGTCACGGG
aroG-2F	CCCGTGACCTGACGAGTAACACGTCCCGTATCGCGGACACAAAA
aroG-2R	GACTCTAGAGGATCCGGTGCCAATGGTGCCTACTATTTGA
aroH-1F	CATGATTACGAATTCAAATCGCGACAGGATCAGTCCTG
aroH-1R	TTCCGCCCCTGTAGGAGCAG
aroH-2F	CTGCTCCTACAGGGGCGGAAATTCAAGGCTTCCTGGGCAGG
aroH-2R	GACTCTAGAGGATCCCGTGGCGAGTGTGTCATAAAACCT
**For Gene Overexpression**	
G-phzC-1F	TACCGGGCCCCCCCTCGAGTTTGAGCACCACTAAAGTTGAAAACAGG
G-phzC-1R	GGCGGCATCCTCCTTAGTTGGG
G-phzC-2F	CCCAACTAAGGAGGATGCCGCCATGGAAGACTTACTGAAACGGGTATTAAGTTG
G-phzC-2R	TGGCGGCCGCTCTAGATCAAAAGGAGGCAAGGGTTGAGGAG
G-aroF-1F	TACCGGGCCCCCCCTCGAGTTTGAGCACCACTAAAGTTGAAAACAGG
G-aroF-1R	GGCGGCATCCTCCTTAGTTGGG
G-aroF-2F	CCCAACTAAGGAGGATGCCGCCATGATGAGCCAACCCTGGAGCC
G-aroF-2R	TGGCGGCCGCTCTAGATCAACGCTTGACCTGTTTCAGGGTC
G-aroG-1F	TACCGGGCCCCCCCTCGAGTTTGAGCACCACTAAAGTTGAAAACAGG
G-aroG-1R	GGCGGCATCCTCCTTAGTTGGG
G-aroG-2F	CCCAACTAAGGAGGATGCCGCCATGGCTGATTTACCGATCAACGACC
G-aroG-2R	TGGCGGCCGCTCTAGATCAGGTACGAACCCGTTTTGGCA
G-aroH-1F	TACCGGGCCCCCCCTCGAGTTTGAGCACCACTAAAGTTGAAAACAGG
G-aroH-1R	GGCGGCATCCTCCTTAGTTGGG
G-aroH-2F	CCCAACTAAGGAGGATGCCGCCATGAACTCGTCCGTATCCGCTCTG
G-aroH-2R	TGGCGGCCGCTCTAGATCAGGCGGAAGCCGGAATGT

## Data Availability

Not applicable.

## Supplementary Materials

The following are available online at https://www.mdpi.com/article/10.3390/biology11010086/s1, Table S1: Comparative analysis of PhzC in PCN producing and Non-PCN producing strains, Table S2: The original data of relative quantification PCR, Table S3: The original data of absolutequantification PCR, Table S4: Calculation of the copy numbers of target gene.
