# Supplementary material for: Uncovering the Role of PhzC as DAHP Synthase in Shikimate Pathway of Pseudomonas chlororaphis HT66
Source: Biology (Basel). 2022 Jan 6;11(1):86. doi: 10.3390/biology11010086 (PMC8772962; doi:10.3390/biology11010086)
Supplement: Supplementary file 1 [file biology-11-00086-s001.zip › biology-1522625-supplementary.pdf]

Table S1: Comparative analysis of PhzC in PCN producing and Non-PCN producing strains

Strain	Phenazine synthesis	Identity
P. chlororaphis GP72	PCA, PCN	96.8%
P. aeruginosa PAO1	PCA	70.5%
P. fluorescens	PCA	93.25%
P. synxantha	PCA	90.25%
P. khavaziana	phenazine	89%
P. libanensis	phenazine	89.8%
P. orientalis	phenazine	89%
E. coli	None	/
C. glutamicum	None	/
S. cerevisiae	None	/
T. reesei	None	/

Table S2: The original data of relative quantification PCR

Sample	*phzC* ct	Sample	*phzE* ct	Sample	*rpoD* ct
12h	16.81091309	12h	16.79423332	12h	17.04684067
12h	16.86725426	12h	16.89256859	12h	17.03855515
12h	16.80355049	12h	16.95059204	12h	17.02434082
24h	16.84362793	24h	16.73035431	24h	16.93395424
24h	16.7544899	24h	16.73167038	24h	16.97888756
24h	16.67251205	24h	16.7148571	24h	16.95400467
36h	16.6044445	36h	16.77412987	36h	17.2119751
36h	16.63935852	36h	16.81188011	36h	17.17531586
36h	16.61040688	36h	16.84941483	36h	17.07945633
48h	17.03374863	48h	17.16329956	48h	17.1572876
48h	17.11420059	48h	17.28756638	48h	17.20553017
48h	17.03970909	48h	17.34780693	48h	17.33313942
A	18.13857574	A	18.29229279	A	18.46237946
A	18.10838699	A	18.2429409	A	18.38371086
A	18.12413216	A	18.25054169	A	18.20348434
+1g/L	16.88474846	+1g/L	17.07390556	+1g/L	17.45089149
+1g/L	16.93598175	+1g/L	17.11664963	+1g/L	17.35134048
+1g/L	16.96905403	+1g/L	17.27592087	+1g/L	17.23365784
+2g/L	17.44518661	+2g/L	17.48888779	+2g/L	17.3517704
+2g/L	17.44421959	+2g/L	17.3073864	+2g/L	17.2923317
+2g/L	17.47684097	+2g/L	17.22134972	+2g/L	17.18089828

Table S3: The original data of absolute quantification PCR

Sample	*phzC*	*aroF*	*aroG*	*aroH*	*phzE*
SD1	8.74	9.5	10.13	10.51	12.33
SD2	12.78	13.47	12.79	13.16	15.3
SD3	16.94	16.01	16.6	17	19.41
SD4	21.08	19.89	19.95	20.49	23.15
SD5	24.64	22.89	24.2	23.96	26.88
SD6	28.85	26.32	27.46	27.74	30.29
WT	26.2	24.83	26.61	24.2	35.56

Table S4. Calculation of the copy numbers of target gene

**Plasmid**[a]	**Concentration**			**Mole**	**Plasmid (bp)**		**Copy/μL**
dsDNA	ng/μL[b]	qPCR ng/μL[c]	g/μL[d]	copy[e]/mol	bp[f]	Da(g/mol)[g]	Plasmid[h]/μL
A	10	0.2	2E-10	6.02E+23	5868	3872880	4.67E+07

a: Plasmid number, b: Standard concentration, c: Standard concentration after dilution (ng/μL), d: Standard concentration after dilution (g/μL), e: Mole, f: Size of plasmid containing target gene, h: Number of plasmids (copy/μL or copy/g cDNA)

Dilution standard: 10-fold serial dilution according to the above-mentioned copy number concentration. When detecting by qRT-PCR, the loading volume of the standard is 5 μL. Standard copy number (X0) calculation method: Ct=-klogX0+b (k represents the slope of the standard curve, b represents the intercept of the standard curve).

In Figure 6A, the gene copy number of *aroF*, *aroG*, *aroH*, *phzC* and *phzE* was absolutely quantified according to a previous report (Bustin 2000).

1) Cloning the target gene into plasmid pBBR to prepare a standard curve.

2) Drawing a standard curve with a standard gene of known copy number.

3) Calculating the initial concentration of the sample from the standard curve by measuring the Ct value of the unknown sample, so as to realize the calculation of the absolute expression amount of the target gene copy number (Table S4).
